# Green Synthesis of Hexagonal Hematite (α-Fe_2_O_3_) Flakes Using Pluronic F127-Gelatin Template for Adsorption and Photodegradation of Ibuprofen

**DOI:** 10.3390/ma14226779

**Published:** 2021-11-10

**Authors:** Maria Ulfa, Didik Prasetyoko, Hasliza Bahruji, Reva Edra Nugraha

**Affiliations:** 1 Chemistry Education Study Program, Faculty of Teacher Training and Education, Sebelas Maret University, Jl. Ir. Sutami 36A, Surakarta 57126, Indonesia; 2 Department of Chemistry, Faculty of Science, Institut Teknologi Sepuluh Nopember, Keputih, Sukolilo, Surabaya 60111, Indonesia; didik.prasetyoko@gmail.com; 3 Centre of Advanced Material and Energy Sciences, University Brunei Darussalam, Jalan Tungku Link, Gadong, Bandar Seri Begawan BE1410, Brunei; hasliza.bahruji@ubd.edu.bn; 4 Department of Chemical Engineering, Faculty of Engineering, Universitas Pembangunan Nasional “Veteran” Jawa Timur, Surabaya 60294, Indonesia; revaedranugraha@gmail.com

**Keywords:** α-Fe_2_O_3_, hematite, gelatin, F-127, hexagonal-flake, adsorption, ibuprofen

## Abstract

Hematite (α-Fe_2_O_3_) with uniform hexagonal flake morphology has been successfully synthesized using a combination of gelatin as natural template with F127 via hydrothermal method. The resulting hematite was investigated as adsorbent and photocatalyst for removal of ibuprofen as pharmaceutical waste. Hexagonal flake-like hematite was obtained following calcination at 500 °C with the average size was measured at 1–3 µm. Increasing the calcination temperature to 700 °C transformed the uniform hexagonal structure into cubic shape morphology. Hematite also showed high thermal stability with increasing the calcination temperatures; however, the surface area was reduced from 47 m^2^/g to 9 m^2^/g. FTIR analysis further confirmed the formation Fe-O-Fe bonds, and the main constituent elements of Fe and O were observed in EDX analysis for all samples. α-Fe_2_O_3_ samples have an average adsorption capacity of 55–25.5 mg/g at 12–22% of removal efficiency when used as adsorbent for ibuprofen. The adsorption capacity was reduced as the calcination temperatures increased due to the reduction of available surface area of the hexagonal flakes after transforming into cubes. Photocatalytic degradation of ibuprofen using hematite flakes achieved 50% removal efficiency; meanwhile, combination of adsorption and photocatalytic degradation further removed 80% of ibuprofen in water/hexane mixtures.

## 1. Introduction

Hematite has been widely investigated as adsorbent for removal of dye, pharmaceutical compound, heavy metal, and industrial waste [1,2,3,4]. Hematite is also referred to as α-Fe_2_O_3_, which is one of the most thermodynamically stable types of iron oxide because of the strong interaction between the electrons [5]. Hematite structure resembles corundum, in which the Fe^3+^ cations are connected to the octahedral oxygen via the covalent bonds in a hexagonal closed packing crystal system [4]. Hematite was reported to have high adsorption performance and stability, and to be easy and cheap to synthesize [1,6,7,8]. The surface area of hematite was reported between 10–90 m^2^/g, thus showing ability as adsorbent in the removal of cephalecin, acetylsalicylic acid, congo red, and heavy metals [2,3]. The hydrophilicity of hematite is reasonably high, beneficial for the adsorption of many organic molecules in water [9]. In addition, the presence of Fe^3+^ ion and the surface OH group formed chemical and physical interactions with organic molecules [2,10]. Hematite was reported to prevent flotation when used as adsorbent for oleate [4]. Antisteroidal agent waste, such as ibuprofen, polluted water and land when discharged from the hospital drainage untreated [11]. Removal of ibuprofen was carried out via high temperature decomposition or with the use of solvent [11,12,13]. The used of solvent to remove ibuprofen is less environmentally friendly; meanwhile, the high temperature decomposition method requires a large amount of energy. Apart from its performance as adsorbent, hematite has 2.1–2.3 eV band gap which is suitable as photocatalyst for photodegradation of pollutants [14]. The conduction band of hematite is composed of empty orbitals in the *d* band of Fe^3+^ and the valence band from the 3d crystal plane that was filled with Fe^3+^ from the formation of 2p non-bonding orbitals [15]. The semiconductor property causes hematite to be widely used as photocatalysts, pigments, and gas sensors [8,9,16].

Hematite is naturally available in abundance, non-toxic to the environment, and its chemical activity is greatly influenced by the multiple oxidation states. Hematite can be synthesized using arc-discharge, micro-emulsion, thermal decomposition, hydro-thermal synthesis, ball milling, sol–gel, electrolysis, and co-precipitation methods [6]. Another method that received increasing attention is preparation of iron oxide using a green templating method. The use of synthetic structure directing agents such as P-123, F-127, cetyl trimethylammonium bromide, and cetyl trimethylammonium chloride [17,18,19,20,21] can be minimized by replacing the template with biodegradable natural reagents. Green synthesis is also beneficial in minimizing the production of residual waste from utilization of hazardous chemical reagents [6,22]. Green synthesis using plant extract from green tea, kurkuma, and lantana fir leaves produced hematite with rod, hexagonal, cone cube, and flake structures [6,23]. However, a high concentration of plant extract was required for rearrangement of the molecules to form uniform structure. The plant extract was unable to direct the formation of pores and prone to reduction lead to deactivation of molecular rearrangement process. Therefore, stabilization of plant extract during the synthesis is required either via pH variation, temperature, or time regulation, and some reactions required nitrogen gas to increase the stability. In addition, plant extract also formed a residual by-product on the synthesized material that can interfere with the characterization and the application [24,25,26,27]. The use of plant extract can be replaced using extract from animal such as gelatin. Gelatin derived from the hydrolysis of animal skin and bone waste has a fairly stable amine group that can form a strong affinity with precursor as pore directing agent in the molecular rearrangement process [28,29].

We have previously reported that the combination of gelatin with block copolymer F127 produced carbon materials with variation of shapes such as carbon foam worm-holes and mesoporous carbon microspheres with high surface area up to 220 m^2^/g [19,22]. Since green synthesis of hematite requires a high stability of template that promises a high regeneration power, this research aimed to synthesize hematite using a combination of gelatin and F127 as structure directing agents. The structural properties of hematite were investigated by variation of calcination temperatures between 500–700 °C. We observed transformation of flake-like morphology to cubic structure with increasing the calcination temperatures. The α-Fe_2_O_3_ flakes were utilized as adsorbent and photocatalyst for removal of ibuprofen as pharmaceutical waste.

## 2. Experiment

### 2.1. Preparation of Hexagonal Flake-Like Hematite (α-Fe_2_O_3_) by Gelatin Template

Ferric chloride (FeCl_3_, MW 162.20, reagent grade 97%), Pluronic F127, HCl, gelatin and ibuprofen (2-[4-(2-methylpropyl) phenyl] propanoic acid) were obtained from Sigma Aldrich and used without prior pretreatment. For the synthesis of hematite flake, Pluronic F127 powder was added into HCl solution and stirred in room temperature for 24 h. The gelatin powder was then added into the mixture followed by ferric chloride. The mixture was stirred until a homogeneous solution was formed. The weight ratio of the synthesis materials was used as follows: 1 Pluronic F127: 0.05 gelatin: 5 Ferric chloride: 0.015 HCl. The resulting homogeneous mixture was then poured into an autoclave with Teflon liner and heated at 100 °C for 24 h. The solid was filtered and calcined at 500 °C for 5 h. Finally, the black powder was washed, filtered, and dried overnight at 100 °C. The temperature of calcination was increased to 600 °C and 700 °C to obtain hematite samples which were labeled as Fe_2_O_3_-G-xC, where ‘x’ is the calcination temperature. 

### 2.2. Characterization

The crystalline phase of hematite was investigated using X-ray diffraction (XRD). XRD pattern was obtained by Philips X’pert XRD (Surabaya, Indonesia) instrument with Cu Ka radiation with a step size of 0.04° and counting time of 10 s. The data were recorded in the 2θ between 5–80°. The crystallinity of iron oxide was calculated based on the ratio of the area of the crystalline peaks to the total area under all peaks as shown by Equation (1) [30].

(1) $$\mathrm{Crystallinity}=\frac{\mathrm{Total}\;\mathrm{area}\;\mathrm{of}\;\mathrm{crystalline}\;\mathrm{peaks}\;}{\mathrm{Total}\;\mathrm{area}\;\mathrm{of}\;\mathrm{all}\;\mathrm{peaks}}$$

The morphology of the samples and elemental composition were analyzed using scanning electron microscopy (SEM) ZEISS EVO MA (Zeiss, Surabaya, Indonesia) and coated by Pd/Au and energy dispersive X-ray spectroscopy (EDS) and alsoTransmission Electron Microscope (TEM HT7700, 120kV, Bandung, Indonesia). Surface area of the sample was measured using nitrogen as adsorbate at P/P0–0.99 by the BET method (NOVA instruments^©^ 1994–2010, Quantachrome Instruments version 11.0-Semarang, Indonesia. The BJH and SF method were used to determine the size of mesopores and micropores, respectively. The pore size distribution curve was derived from the desorption branch using the Barrett–Joyner–Halenda (BJH) model. The functional group of the materials were characterized through Fourier transform infrared spectroscopy (Nicolet 6700 FTIR instrument, Thermo Fisher Scientific, Surakarta, Indonesia).

### 2.3. Ibuprofen Adsorption

To investigate the adsorption profiles of hematite, ibuprofen solution was prepared in water–hexane (1:1 v/v) at 100 mg L^−1^ of initial concentration. The concentration of ibuprofen was monitored using UV–vis spectroscopy at 272 nm wavelength. The calibration was carried using ibuprofen standard solutions with concentration of 5–100 mg L^−1^. 50 mg of the resulting hematite were added into 50 mL of ibuprofen solution and stirred at room temperature for 30 min at neutral pH. 3 mL of ibuprofen solution were sampled at 5 min intervals and separated using membrane filter followed by UV–vis measurement. The analysis was carried out until the adsorption reached equilibrium. The adsorption was repeated up to 60 min to monitor the stability of the ibuprofen adsorption on hematite. UV–vis analysis was performed using U-2000 Hitachi Japan. The calculation of the adsorption capacity and the adsorption kinetics were based the previous research [19] as shown in the following equation

(2) $$q_{e}=\left(Co-Ce\right)\left(\frac{v}{w}\right)$$

where *q_e_* (mg/g) is the adsorption capacity in equilibrium, while Co,Ce,v, and w are the initial concentration (mg/L), concentration of ibuprofen in equilibrium (mg/L), volume of ibuprofen (L), respectively, and adsorbent weight (mg) Co and Ce. Meanwhile, the adsorption kinetics follows the pseudo first order by Lagergren equation

(3) $$\ln(q_{e}-q_{t})=\ln q_{e}-k_{1}t$$

and the pseudo second order equation by Ho McKay follows

(4) $$\frac{t}{q_{t}}=\frac{1}{k_{2}{\left(q_{e}\right)}^{2}}-\frac{1}{q_{e}}\;t$$

The linearity value of R^2^ plotted t vs. $\ln(q_{e}-q_{t})$ dan t vs. $\frac{t}{q_{t}}$ will be used to determine the appropriate kinetics for the adsorption of ibuprofen using iron oxide in this study.

### 2.4. Photocatalytic Degradation of Ibuprofen

**Figure 1:**
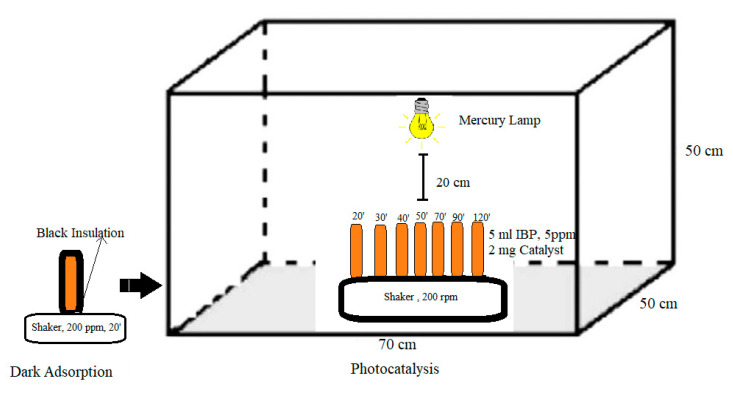
Schematic representation of photocatalytic reactor setup for degradation of ibuprofen. The black box was used to prevent light from surrounding for dark adsorption reaction.

Photocatalytic activity of iron oxide in the degradation of ibuprofen was determined using 125 W UV light at wavelength of 265 nm in reactor setup shown in Figure 1. Ultraviolet irradiation by mercury lamp (Philip HPL-N 125W/542 E27) was monitored using a spectroradiometer (Lumen spectroradiometer-Stellarnet-20 mA) with the light intensity being determined at 166 mW/cm^2^. Photocatalytic degradation was carried out by adding 5 mL of ibuprofen solution (5 mg/L) and 2 mg of hematite into a quartz tube and irradiated with UV light at different time intervals such as 20, 30, 40, 50, 70, 90, and 120 min under continuous stirring. Three separate experiments were carried out: in dark, under immediate light irradiation, and 20 min dark adsorption prior to UV irradiation. The percentage of ibuprofen degradation was calculated as

Removal = ((*Co* − *C_t_*)/*Co*)) × 100 (5)

where *Co* and *C_t_* represent ibuprofen concentration in the solution before and after the UV irradiation respectively.

## 3. Results and Discussion

### 3.1. XRD Analysis

**Figure 2:**
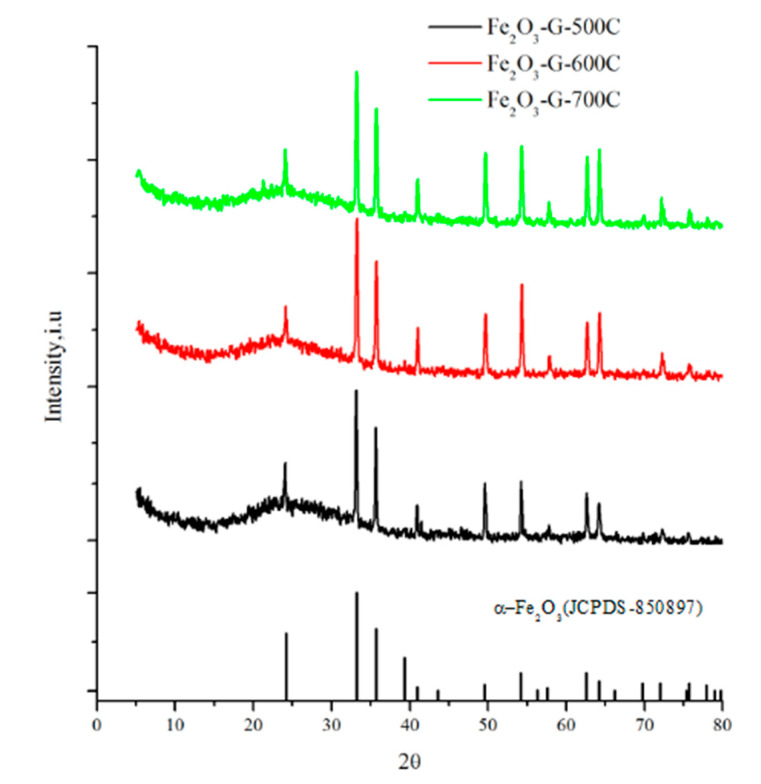
XRD of hematite synthesized with F127-gelatin after calcination at black 500 °C, red 600 °C, and green 700 °C for 5 h.

Figure 2 shows the X-ray diffractogram of hematite synthesized using F127-gelatin as structure directing agent (Fe_2_O_3_-G) after calcination at 500 °C, 600 °C, and 700 °C. The main peaks for α-Fe_2_O_3_ (JCPDS-850897) were observed at 24.08°, 33.10°, 35.56°, 40.96°, 49.52°, 54.24°, and 57.82° with the respective miller field of (012), (104), (311), (113), (024), (116), and (018). The XRD of the synthesized hematite is also in accordance with the literature [7,31]. When the calcination was increased from 500 °C to 700 °C, there were no significant differences on the position of hematite peaks, suggesting the stability of hematite. However, the peak at 33.10° (104) and 40.96° (113) which were exclusive to α-Fe_2_O_3_ showed a higher intensity as the calcination temperatures increased to 700 °C. The result suggests the conversion of γ-Fe_2_O_3_ to α-Fe_2_O_3_. At 500 °C, the XRD data also showed a broad peak at 20–30° due to the formation amorphous structure in hematite [32,33]. The amorphous structure was suggested as the result of incomplete decomposition of F127 and gelatin, leaving residual amorphous carbon. However, following calcination at 600 °C and 700 °C, the broad amorphous peaks were slightly reduced due to the elimination of residual amorphous carbon. The result was further supported by EDX data (Table 1) which showed the samples contained 3.18% and 0.75% of carbon after calcination at 500 °C and 600 °C, respectively. However, at 700 °C, carbon was completely removed from the sample resulting in the formation of high purity hematite. The crystallinity of iron oxides was calculated based on the ratio of the area of the crystalline peaks over the total area including the broad amorphous peak. Increasing the calcination temperatures from 500 °C to 700 °C significantly enhanced the crystallinity from 48.31% to 63.19% (Table 1).

### 3.2. SEM Analysis

**Figure 3:**
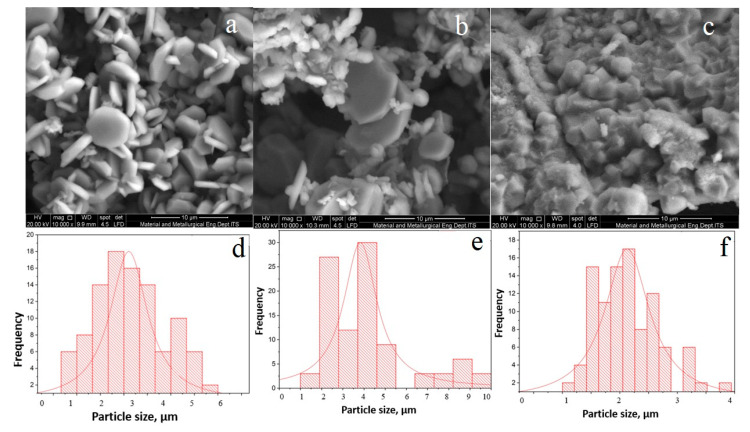
SEM and histogram of particle size distribution of iron oxide synthesized after calcination for 5 h at 500 °C (**a**,**d**), 600 °C (**b**,**e**), and 700 °C (**c**,**f**).

**Figure 4:**
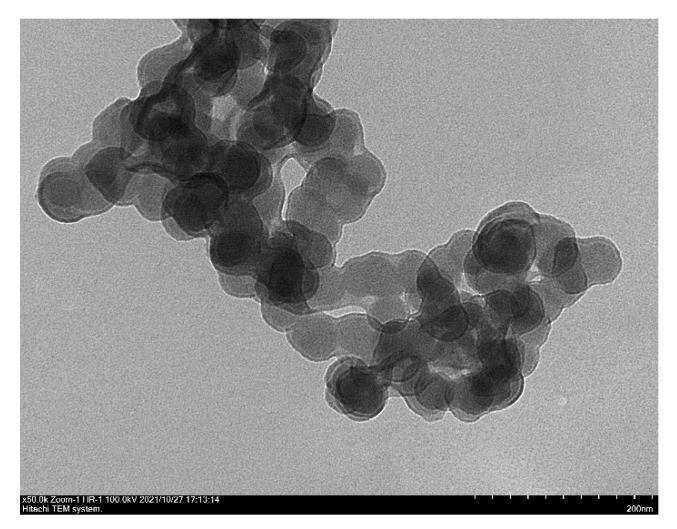
TEM analysis of iron oxide after calcination for 5 h at 500 °C.

Figure 3 showed the SEM analysis of hematite calcined at 500 °C, 600 °C, and 700 °C together with the particle size distribution histograms. As evidence from EDX analysis, calcination at high temperatures were important for removal of carbon residues originated from decomposition of the template. SEM analysis indicated significant structural changes following calcination at high temperatures. At 500 °C, hematite showed the formation of uniform flake-like hexagonal shape. The particle size histogram exhibited that a narrow distribution with the average size was determined between 1–3 µm. When the calcination temperature was increased to 600 °C, the uniformity of the flake-like hexagonal structure was deteriorated, with apparent structural disintegration to form smaller aggregates. Nevertheless, the flake-like hexagonal structures were still visible and the size was increased to 4–5 µm. The presence of F127 and gelatin as template was responsible to enhance the structural stability of iron oxide in order to maintain the flake-like hexagonal structure after calcination at 600 °C. Following calcination at 700 °C, transformation from flake-like hexagonal structure to cube was observed, and the size reduced to 1–2 µm. In the synthesis of iron oxide, calcination at 500 °C is important for the formation of bonds between the iron oxide grains [34,35]. However, calcination at 700 °C was important to remove carbon impurities, although the morphology was transformed from hexagonal flake-like structure into cubic structure. TEM analysis was also carried out on iron oxide after calcination for 5 h at 500 °C (Figure 4). The TEM image showed a uniform morphology of iron oxide particles that were intercalated to form a long network. 

Characterization results obtained from XRD, SEM, and TEM analysis provided insight into the stability of α-Fe_2_O_3_ morphology. Although α-Fe_2_O_3_ was calcined at 500 °C in order to remove the template during synthesis, the flake-like structures were retained and stable up to 600 °C. The ability to direct the morphology strongly relied on the presence of F127 and gelatin to form a stable micellar structure [22]. The OH functional groups in the copolymer block F127 and NH on the gelatin have a strong affinity for interacting with the iron precursor, so that these two molecules were able to direct the structure of the material [22]. Gelatin consists of carboxyl, amino, and hydroxyl groups acting as sites to form coordination with metal ionic species [29]. Fe^2+^-gelatin complex formation stabilized iron from dissolution [36]. However, due to the stability of block copolymer F127 and gelatin interaction with the elemental iron, decomposition at 700 °C is crucial to fully remove the residual carbon. The presence of carbon residue was still observed after calcination at 500–600 °C suggesting the strong interaction between iron and gelatin to maintain flake structures. However, as the temperature increased to 700 °C, the transformation of flake-like structure to cubic morphology occurred together with the removal of carbon impurities. The results further implicated the role of gelatin in improving the stability of α-Fe_2_O_3_.

### 3.3. N_2_ Adsorption Analysis

**Figure 5:**
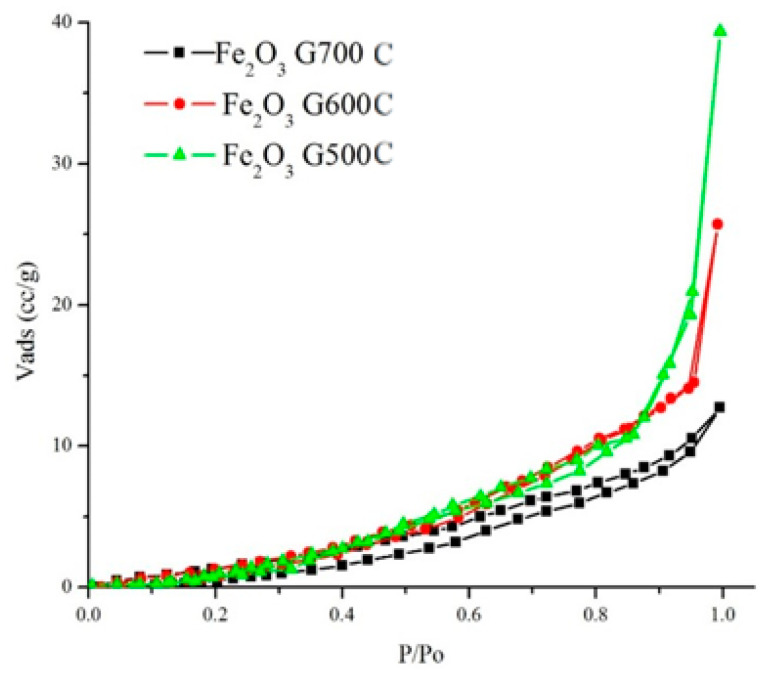
Nitrogen adsorption–desorption analysis of iron oxide synthesized using F127-gelatin after calcination at a. 500 °C, 600 °C, and 700 °C for 5 h.

Figure 5 showed the nitrogen adsorption isotherm of iron oxide following calcination in air at 500 °C, 600 °C, and 700 °C for 5 h. The N_2_ isotherm showed a typical type-III isotherm with the H1 hysteresis loop without capillary condensation which is the characteristic of a non-porous material. The specific surface area of α-Fe_2_O_3_ oxides after calcination of 500 °C was 49 m^2^/g. The surface area was significantly reduced to 16 m^2^/g following calcination at 600 °C and further reduced to 7 m^2^/g when calcined at 700 °C. The pore volume analyzed using BJH method indicated the reduction from 0.161 cc/g to 0.030 cc/g when α-Fe_2_O_3_ was calcined from 500 °C to 700 °C. The surface area and the pore volume summarized in Table 2 were significantly influenced by the calcination temperatures, due to the transformation of crystallite structure of iron oxide which is in line with previous result [31,32,33,34,35].

**Figure 6:**
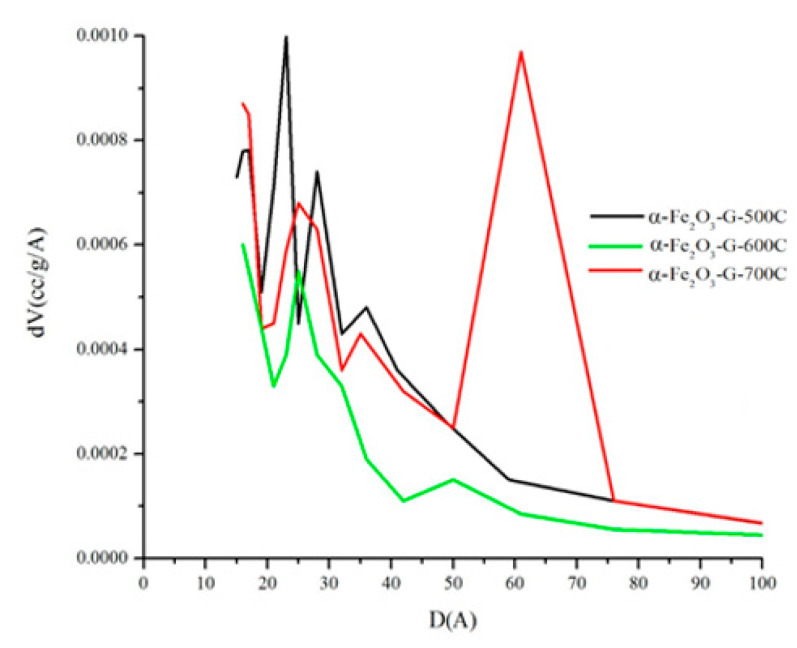
Pore size distribution of iron oxide synthesized with F127-gelatin after calcination for 5 h.

Although the synthesized hematite showed the typical non-porous isotherm, the presence of hysteresis loop at high pressures indicated the formation of macropores originated from the interparticle interaction. The plot of pore size distribution in Figure 6 showed the average pore diameter was increased at high calcination temperatures from 37 Å when calcined at 500 °C to 83 Å when calcined 700 °C. The increase of pore size is presumably due to the decomposition of carbon from the F127 and gelatin copolymer composite that previously occupied the vacant sites between the iron oxide particles. The decomposition of carbon at high temperatures also increased the possibility for the formation of pores as new cavities due to the loss of macromolecules carbon during its decomposition. From the SEM analysis, the macropore or mesopore region cannot be determined because SEM produced a three-dimensional image of the iron oxide aggregates. Therefore, the porosity was determined by using N_2_ adsorption–desorption isotherm plot as well as the pore size distribution plot.

### 3.4. Ibuprofen Removal via Adsorption

**Figure 7:**
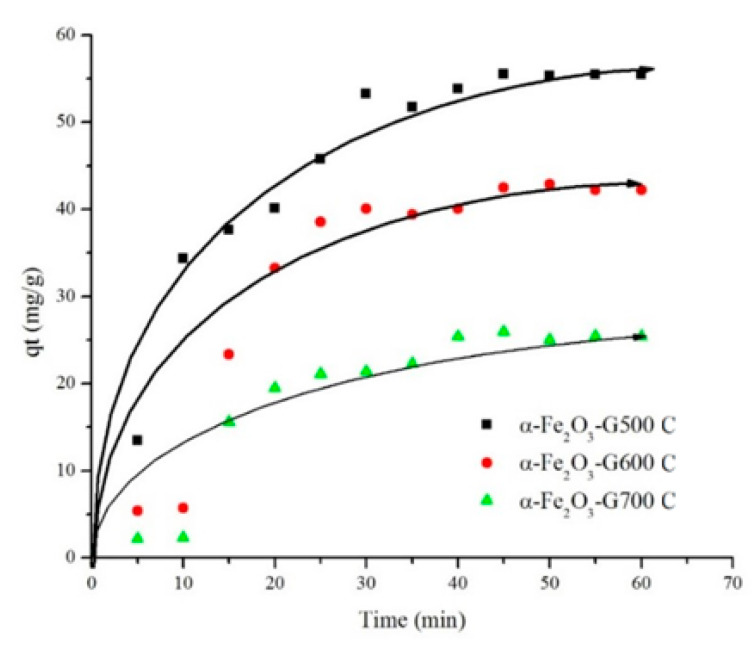
Effect of contact time on ibuprofen adsorption (50 mg/L) while using hematite after calcination at 500 °C, 600 °C, and 700 °C.

Figure 7 showed the adsorption of ibuprofen on hematite within 60 min of contact. The amount of adsorbed ibuprofen was analyzed on the resulting iron oxides after calcination at 500 °C, 600 °C, and 700 °C. The results showed rapid adsorption within the first 30 min, followed by gradual adsorption before reaching equilibrium at 50 min. This was due to the decrease of surface area upon adsorption with ibuprofen, thereby reducing the availability of active binding sites at the saturation point as the adsorption reaching the equilibrium. The maximum adsorption capacity was determined on iron oxide calcined at 500 °C with the adsorption capacity was measured at 55.51 mg/g. When iron oxide calcined at 600 °C was used, the adsorption reduced to 42.12 mg/g and further reduction to 25 mg/g on iron oxide calcined at 700 °C. The removal efficiency was decreased from 22.2% to 11.1% with increasing the calcination temperatures up to 700 °C. The decrease in surface area of iron oxide when calcined at high temperature reduced the number of available sites for ibuprofen adsorption. The adsorption of ibuprofen on α-Fe_2_O_3_-G500 occurs on the expose site consisting of dangling Fe bond which forms an interaction with the OH group in ibuprofen [37]. The adsorption capacity of iron oxide with flake-like morphology as adsorbent for ibuprofen at 55.55 mg/g was higher than adsorption on α-FeOOH at 3.47 mg/g [37], natural O-carboxymethyl-N-laurylchitosan/α-Fe_2_O_3_ at 6–15 mg/g) [38], and composite iron nano adsorbent at 50 mg/g [39]. Furthermore, the adsorption was also higher than different type of adsorbent such as clay at 35 mg/g [40], pine wood at 10 mg/g [41], and graphene oxide nanoplatelets [42].

**Figure 8:**
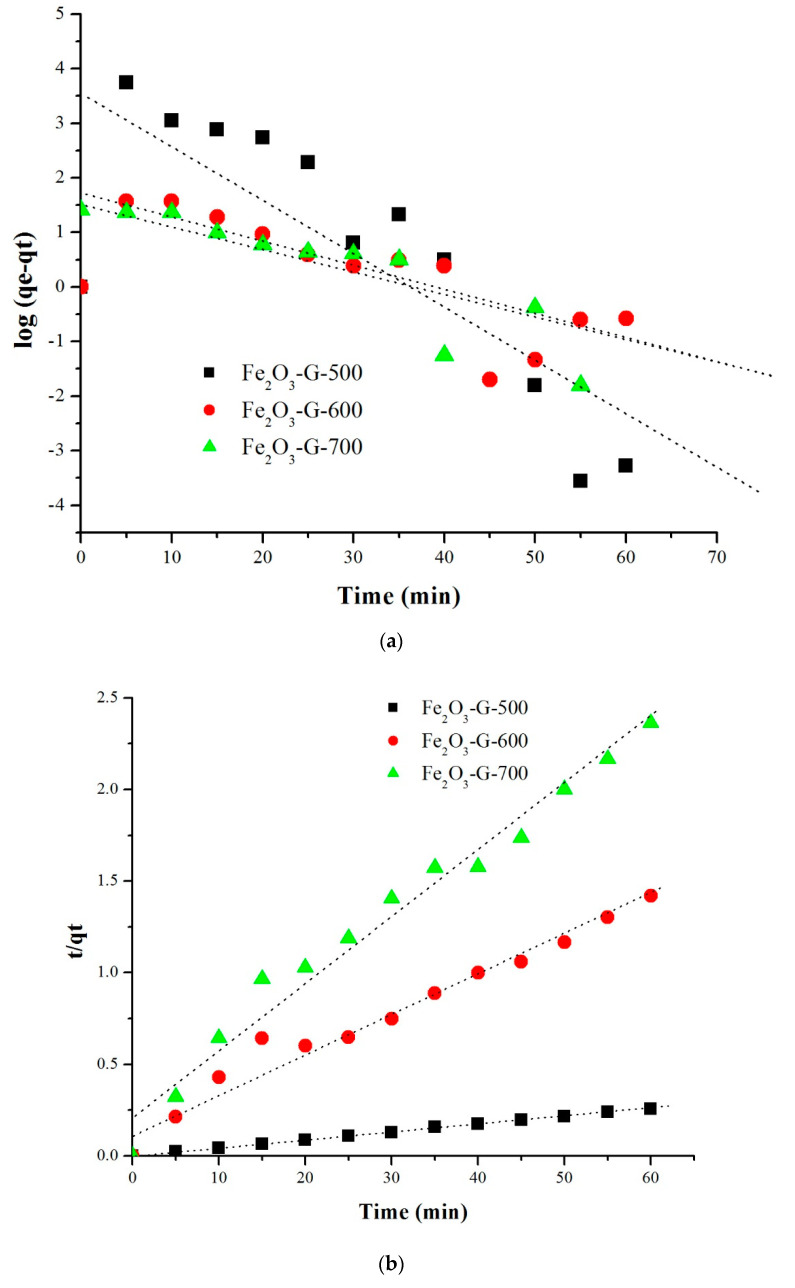
(**a**) Pseudo first order plot and (**b**). Pseudo second order plot of the kinetic of ibuprofen adsorptionusing iron oxide.

Table 3 summarized the kinetic analysis data using the pseudo first order and the pseudo second order equations. The plot between $\ln(q_{e}-q_{t})$ versus t for the pseudo-first-order model (Figure 8a) and *t*/*q_t_* versus *t* plot for the pseudo-second-order model (Figure 8b) were carried out to obtain the regression coefficient value related to linearity (R^2^). The pseudo first order plot showed the R^2^ values for all the sample were within 0.5–0.7. Whereas, for the pseudo second order plot, the R^2^ value of each calcined samples were closed to 1 thus indicating the adsorption of ibuprofen on Fe_2_O_3_-G followed the pseudo-second kinetic model.

**Figure 9:**
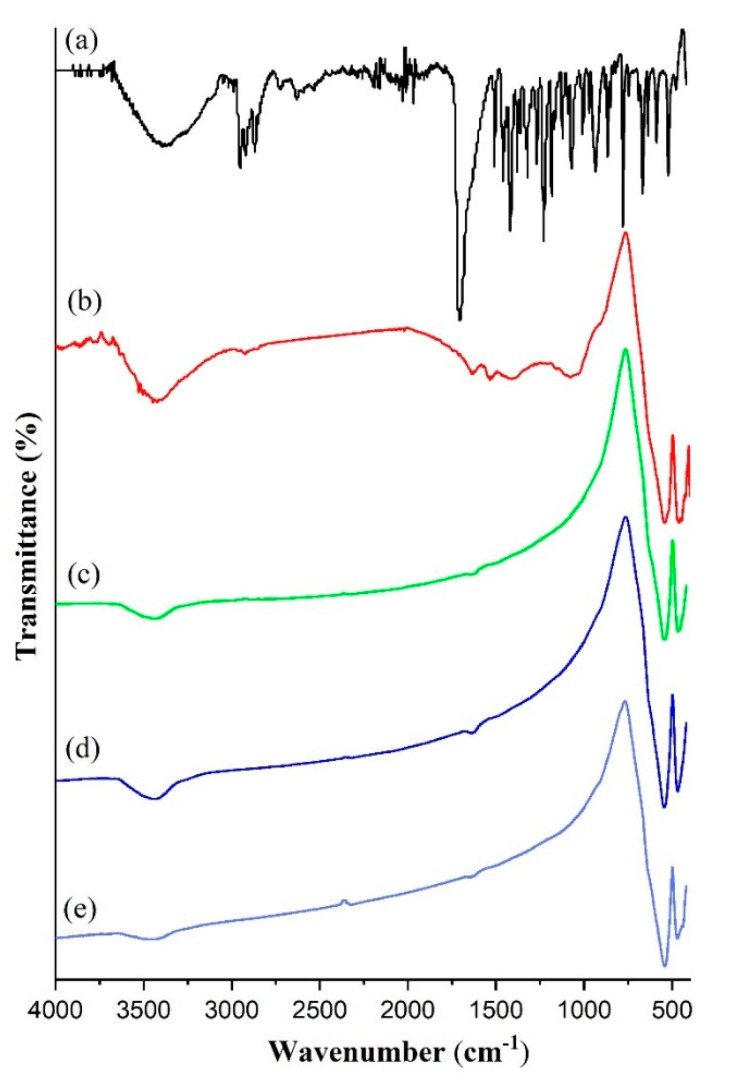
FTIR of (**a**) ibuprofen, (**b**) Fe_2_O_3_-G500 after ibuprofen adsorption and Fe_2_O_3_-G iron oxide synthesized with F127-gelatin after calcination at (**c**) 500 °C, (**d**) 600 °C, (**e**) 700 °C for 5 h (before ibuprofen adsorption).

FTIR analysis was carried out to determine the adsorption of ibuprofen on hematite. Figure 9 showed a broad band between 3000 and 3500 cm^−1^ and small band at 1624 cm^−1^ which indicated the stretching vibration of hydroxyl group or physisorbed water. The strong adsorption on hematite samples at 431 cm^−1^ and 577 cm^−1^ corresponded to the Fe-O bond vibrations [7]. There were no visible changes in the intensity of the adsorption bands for iron oxide samples after calcination up to 700 °C. Figure 8 also showed that ibuprofen has an intense and well-defined infrared band at 1700–2100 cm^−1^ which was associated with the stretching of the C=O carbonyl group of ibuprofen [43]. Following adsorption with ibuprofen on Fe_2_O_3_-G500, the new adsorption bands appeared at 2929 cm^−1^ assigned to C-H vibration of ibuprofen, 1634 cm^−1^ due to C=O vibration, 1527 cm^−1^ corresponded to C=C vibration and 1064 cm^−1^ originated from C-O bond in the alcohol functional group in ibuprofen [44]. The shifting of C=O absorption band at 1709 cm^−1^ of ibuprofen to 1634 cm^−1^ on adsorbed Fe_2_O_3_ implied a weakened C=O bond due to interaction with iron oxide.

It is also worth investigating the crystallinity of hematite using data obtained from FTIR analysis. Based on previous study which reported that through FTIR, the crystallinity index can be determined by comparing the absorbance at the peak containing the crystalline skeleton that formed the functional group [39,40,45]. In this sample, the main constituent of iron and oxygen in the main functional group Fe-O appeared at 431 cm^−1^ and 577 cm^−1^. The ratio between the intensity of these peak was calculated to represent the changes on crystallinity of iron oxide following calcination at high temperatures. The results in Table 4 showed the I_431_/I_577_ ratios increased from 1.030 to 1.18 when calcined at 500 °C and 700 °C, respectively (as attached in the supplementary file Table S1). The analysis further supported significant changes of iron oxide crystallinity with increasing calcination temperatures. The differences of crystallinity between hematite calcined at 500 °C and 700 °C from FTIR and XRD data were determined at 12.6% and 23%, respectively.

**Figure 10:**
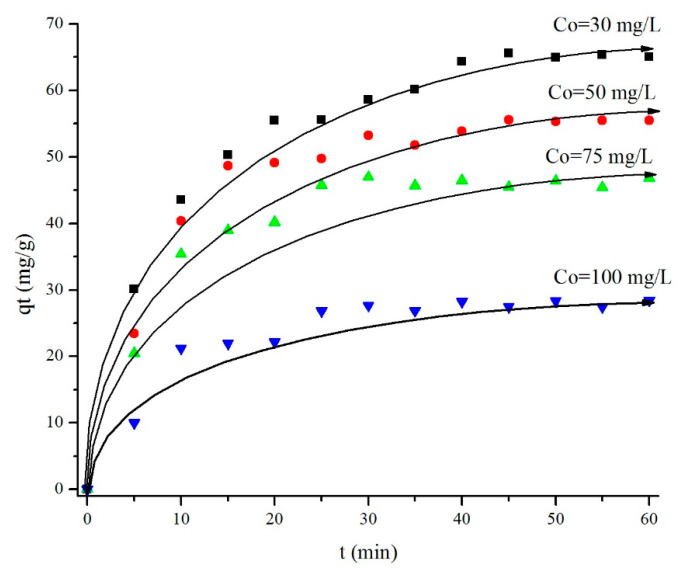
Effect of initial concentration of ibuprofen (Co) during adsorption using Fe_2_O_3_-G-500.

The effect of ibuprofen concentration on adsorption capacity was investigated using Fe_2_O_3_-G-500 at 30 mg/L, 50 mg/L, 75 mg/L, and 100 mg/L. Figure 10 showed that the adsorption capacity decreased gradually from 64 mg/g to 28 mg/g with increasing concentration of ibuprofen. This phenomenon is probably due to the presence of concentration gradient that causes a change in the driving force of ibuprofen into the adsorbent [46]. Furthermore, the surface area of iron oxide is relatively smaller than that of most adsorbent, thus unable to adsorb a high concentration ibuprofen resulting in the decrease of adsorption capacity [47]. The decrease of adsorption sites saturated with ibuprofen molecules is predicted to have a strong influence in decreasing the adsorption capacity by two times, as observed in 50 mg/L and 100 mg/L of ibuprofen concentrations [48]. The percentage of removal when using a high concentration solution at 100 mg/L was observed in only 18.7%, in agreement with previously reported studies that suggested the available active sites were already saturated by ibuprofen moieties [49]. Apart from that, the increase of ibuprofen concentration in this study will cause a decrease in concentration as the results of previous research with an initial concentration of 25–75 mg/L resulting in a decreased adsorption capacity from 12 mg/g to 6.5 mg; 7/g with an adsorbent weight of 75 mg [50]. Therefore, we conclude that under neutral conditions, the higher initial concentration of ibuprofen will cause a decrease in adsorption capacity due to the decrease in the active site due to being covered by the number of interacting molecules.

**Figure 11:**
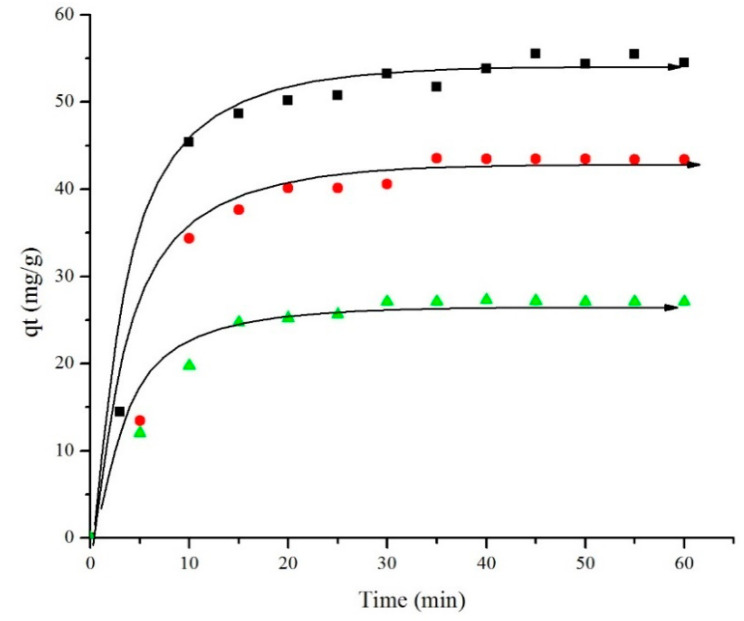
Effect of temperature at room temperature (black square), 35 °C (red circle), and 45 °C (green triangle) during ibuprofen adsorption using Fe_2_O_3_-G-500.with initial concentration 50 mg/L.

Figure 11 illustrates the effect of temperature on the adsorption capacity of ibuprofen while using Fe_2_O_3_-G-500 as adsorbent. The adsorbed capacity was reduced from 55 mg/g at room temperature to 22 mg/g at 45 °C within 60 min. The decrease in adsorption capacity at high temperature implied the reduction of physical interaction between ibuprofen and the iron oxide active site. High temperature also increased the diffusion rate of ibuprofen from the surface of adsorbent and improved repulsion between the adsorbed ibuprofen molecules creating a steric barrier [48,49,50]. In general, the results showed that temperature is an important parameter to control the efficiency of ibuprofen removal.

### 3.5. Photocatalytic Degradation of Ibuprofen

**Figure 12:**
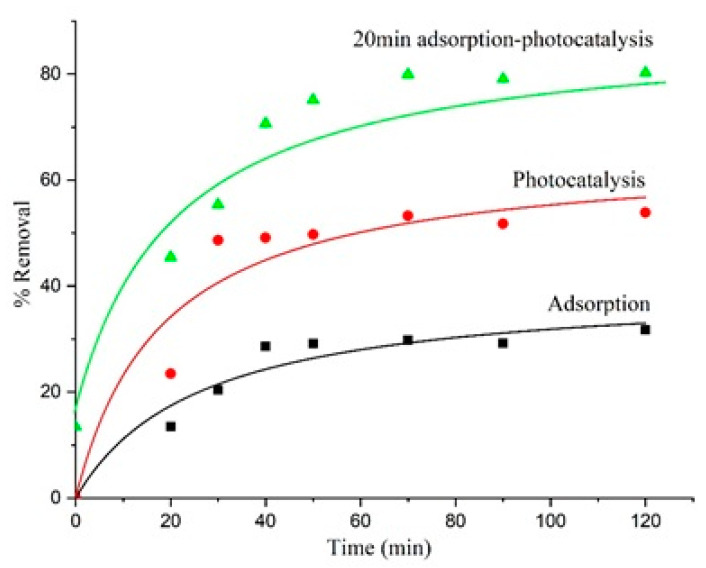
Photocatalytic removal of ibuprofen using Fe_2_O_3_-G-500. Three sets of experiments were conducted to show the efficiency of hematite in photodegradation of ibuprofen; red immediate UV light irradiation for 120 min; black dark adsorption; and green 20 min dark adsorption followed by UV irradiation for 120 min.

Optimization of adsorption condition only achieved 22% of ibuprofen removal on α-Fe_2_O_3_. The low surface area and non-porosity of iron oxide may restrict the number of available sites for ibuprofen adsorption. Since iron oxide has a band gap of energy at 2.3 eV that can utilize photon energy up to 600 nm [51], its activity as a photocatalyst was evaluated for degradation of ibuprofen. Figure 12 shows the effect of UV-light irradiation on the removal of ibuprofen using α-Fe_2_O_3_-G500. Three sets of experiments were conducted; in dark to represent adsorption, immediate light irradiation, and light irradiation after 20 min of adsorption (Figure 1). It is apparent that photocatalytic degradation of ibuprofen using α-Fe_2_O_3_-G500 increased the removal efficiency up to 53%. When compared with removal without UV light (dark adsorption only), the removal of ibuprofen only reached 28%. However, the combination of adsorption followed by UV irradiation increased the ibuprofen removal up to 80%.

The activity of iron oxide as photocatalyst significantly improved the removal of ibuprofen via photodegradation. Preliminary adsorption prior to irradiation has optimized the interaction between ibuprofen and iron oxide. The photodegradation process can take place by direct reaction of hydroxyl radical with the adsorbed ibuprofen on the surface of photocatalysts (Equations (6)–(9)). This process occurs faster due to efficient charge transfer from Fe_2_O_3_ surface to the adsorbed substrate. In photocatalytic degradation without prior adsorption, the photogenerated electron and hole formed mobile OH^•^ radical in the solution via superoxide radicals intermediate (Equations (10)–(13)) [52]. The OH^•^ radical diffuse to oxidize ibuprofen to presumably CO_2_ and H_2_O (Equation (15)). There is a possibility of OH^•^ radical to recombine and released heat.

Adsorption of ibuprofen

C_3_H_18_O_2_ → C_3_H_18_O_2_(a) (6)

Electron excitation

α-Fe_2_O_3_ → α-Fe_2_O_3_ (e^−^ + h^•^) (7)

Electron trapping by surface hydroxyl group

e^−^ + OH(a) → OH^•^(a) (8)

Direct decomposition of adsorbed ibuprofen

C_3_H_18_O_2_(a) + OH^•^(a) → CO_2_ + H_2_O (9)

Electron trapping by dissolved oxygen to form superoxide radicals

e^−^ + O_2_ → O_2_^•−^ (10)

Water dissociation

H_2_O → H^+^ + OH^−^ (11)

Generation of hydroxyl radical

O_2_^•−^ + H^+^ + H_2_O → H_2_O_2_ + O_2_ (12)

H_2_O_2_ → 2OH^•^ (13)

Hole trapping

OH^−^ + h + → OH^•^ (14)

Ibuprofen degradation

OH^•^ + C_3_H_18_O_2_ → CO_2_ and H_2_O (15)

## 4. Conclusions

Iron oxide (Fe_2_O_3_-G) with uniform hexagonal flake morphology has been successfully synthesized using a combination of gelatin as a natural template and F127 as a synthetic template via sol–gel method. SEM analysis showed the formation of uniform hexagonal flake-like structure that was stable after calcination at 500 °C. The transition from hexagonal to cubic structure was observed after calcination at 700 °C. Gelatin as naturally formed polymer showed a potential as structure directing agent for the formation of a highly stable iron oxide with uniform structures. The adsorption capacity of Fe_2_O_3_-G as adsorbent for ibuprofen was determined at 55 mg/g when using the hexagonal flake-like iron oxide. Although the calcination at 700 °C produced a high purity iron oxide, the adsorption capacity and removal efficiency were significantly reduced due to the formation of low surface area cubic crystallites. The efficiency of the adsorption was strongly dependent upon the surface area of iron oxide. The combination of photocatalytic degradation and adsorption for the removal of ibuprofen using iron oxide resulted in the increase of removal efficiency to 80% under UV light irradiation.

## Figures and Tables

**Table 1:** Particle size and elemental composition of hematite at different calcination temperatures.

Sample	^a^ Particle Size (µm)	^b^ Crystallinity (%)	^c^ Elemental Composition%			
			C	O	Fe	Others
Fe_2_O_3_-G-500C	1.0–3.0	48.31	3.18	21.82	74.32	0.68
Fe_2_O_3_-G-600C	4.0–5.0	57.88	0.75	17.17	80.07	2.01
Fe_2_O_3_-G-700C	1.0–2.0	63.19	0	17.15	82.85	0

^a^ determine from SEM analysis. ^b^ determine from XRD analysis. ^c^ determine from SEM-EDX analysis.

**Table 2:** Textural properties of Fe_2_O_3_-G obtained from N_2_ adsorption–desorption analysis.

SAMPLE	S_BET_	V Tot	R(A)
Fe_2_O_3_-G-500C	49	0.166	37.1
Fe_2_O_3_-G-600C	16	0.028	43.3
Fe_2_O_3_-G-700C	7	0.030	83.4

**Table 3:** Kinetics of ibuprofen adsorption using iron oxide sample.

Sample	*Co* (ppm)	*q_e_* Exp (mg/g)	Removal Efficiency, %	Pseudo First Order			Pseudo Second Order		
				*q_e_* Cal (mg/g)	k_1_ (min^−1^)	R^2^	*q_e_* Cal (mg/g)	k_2_ (g·mg^−1^·min^−1^)	R^2^
Fe_2_O_3-_G-500	100	55.51	22.2	419.7	0.0986	0.6514	55.55	0.083	0.999
Fe_2_O_3-_G-600	100	42.12	16.7	390.7	0.0901	0.524	41.66	0.078	0.969
Fe_2_O_3-_G-700	100	25.61	11.1	337.7	0.0926	0.5514	25.28	0.058	0.975

**Table 4:** Summary of crystallinity data calculated from FTIR and XRD analysis.

Sampel	I_431_/I_577_ (FTIR)	Crystalinity % (XRD)	% Ratio Crystalinity (FTIR) ^a^	% Ratio Crystalinity (XRD) ^b^
Fe_2_O_3_-G-500	1.030	48.310	12.649	23.548
Fe_2_O_3_-G-600	1.058	57.880	10.321	8.403
Fe_2_O_3_-G-700	1.180	63.190	0.000	0.000

^a^ Percentage of differences for I _431_/I _577_ of the respective sample relative to Fe_2_O_3_-G-500. ^b^ Percentage of crystallinity of the respective sample relative to Fe_2_O_3_-G-500.

## Data Availability

The data presented in this study are available on request from the corresponding author.

## Supplementary Materials

The following are available online at https://www.mdpi.com/article/10.3390/ma14226779/s1, Table S1: Crystallinity of the intensity ratio at 431 and 577 cm^−1^.
