# Supplementary material for: Green Synthesis of Hexagonal Hematite (α-Fe2O3) Flakes Using Pluronic F127-Gelatin Template for Adsorption and Photodegradation of Ibuprofen
Source: Materials (Basel). 2021 Nov 10;14(22):6779. doi: 10.3390/ma14226779 (PMC8618463; doi:10.3390/ma14226779)
Supplement: Supplementary file 1 [file materials-14-06779-s001.zip › materials-1429593-supplementary.pdf]

materials

MDPI

Supplementary Material

Green synthesis of Hexagonal Hematite (α-Fe_2O_3) Flakes Using Pluronic F127-Gelatin Template for Adsorption and Photodegradation of Ibuprofen

Maria Ulfa [1,*], Didik Prasetyoko [2], Hasliza Bahruji [3] and Reva Edra Nugraha [4]

[1] Chemistry Education Study Program, Faculty of Teacher Training and Education, Sebelas Maret University, Jl. Ir. Sutami 36A, Surakarta 57126, Central Java Indonesia
[2] Department of Chemistry, Faculty of Science, Institut Teknologi Sepuluh Nopember, Keputih, Sukolilo, Surabaya 60111, East Java, Indonesia
[3] Centre of Advanced Material and Energy Sciences, University Brunei Darussalam, Jalan Tungku Link, BE1410 Darussalam, Brunei
[4] Department of Chemical Engineering, Faculty of Engineering, Universitas Pembangunan Nasional "Veteran" Jawa Timur, Surabaya, East Java, 60294, Indonesia
* Correspondence: ulfa.maria2015@gmail.com

Table S1. Crystallinity of the intensity ratio at 431 and 577 $cm^{-1.}$

Sample	I_{431}	I_{577}	I_{431}/I_{577}	%Ratio with Fe_2O_3-G-700
Fe_2O_3-G-600	47,603	46,196	1,030,457	12.6487
Fe_2O_3-G-700	46,102	43,578	1,057,919	10.3207
Fe_2O_3-G-500	40,281	34,146	117,967	0.0000
